# Predicting cell properties with AI from 3D imaging flow cytometer data

**DOI:** 10.1038/s41598-024-80722-6

**Published:** 2025-02-17

**Authors:** Zunming Zhang, Yuxuan Zhu, Zhaoyu Lai, Minhong Zhou, Xinyu Chen, Rui Tang, William Alaynick, Sung Hwan Cho, Yu-Hwa Lo

**Affiliations:** 1 Department of Electrical and Computer Engineering, University of California, San Diego, La Jolla, CA 92093 USA; 2 NanoCellect Biomedical Inc., San Diego, CA 92121 USA

**Keywords:** Biotechnology, Cell biology

## Abstract

Predicting the properties of tissues or organisms from the genomics data is widely accepted by the medical community. Here we ask a question: can we predict the properties of each individual cell? Single-cell genomics does not work because the RNA sequencing process destroys the cell, not allowing us to verify our predictions. To test the hypothesis, we investigate the approach of using AI to analyze single-cell images obtained from a 3D imaging flow cytometer. We analyze the cell image at day zero and make the AI-assisted cell property prediction. The prediction is then examined later when the cells continue to live and develop. Our preliminary results are promising, showing 88% accuracy in predicting cells that will have a high protein expression level. The technique can have strong ramifications and impact on preventive medicine, drug development, cell therapy, and fundamental biomedical research.

**Supplementary Information:**

The online version contains supplementary material available at 10.1038/s41598-024-80722-6.

## Introduction

Unraveling cellular behavior at the single-cell level forms the bedrock of modern cell biology, biotechnology, and therapeutic development^1–4^. To date, research has been focused on studies of the molecular or morphological properties of cells at the time of analysis, limited by the methods of single-cell genomics or proteomics that destroy or seriously disturb the cells^5,6^. While such analysis works well, one potentially important ability all current technologies cannot support is to predict the cell behaviors at the single-cell level.

An interesting question is asked here: what if one can analyze the cell at one time and then predict the cell behaviors in the future? Should we be able to predict the fate of each individual cells accurately, many interesting applications become possible. For example, for manufacturing of monoclonal antibody drugs^7^, the current technique requires selecting single cell derived micro colonies having a high proliferating rate and protein drug production rate. Today people have to place a single cell in each well in well plates for tens of thousands of times, followed by days or weeks of culturing and measurements of the cell properties and finally pick up those single-cell derived micro colonies of high performance for drug production. If one can correctly predict the proliferating rate and drug-protein production rate of each cell from Day Zero, that can yield significant savings in the cost and labor in drug manufacturing. Cell property prediction capabilities can also impact the field of preventive medicine and personalized medicine^8–10^. If one can predict the likelihood of cells that are in sub-healthy or precancerous state to become cancerous, preventive treatments can be administered to lower the risks. In these cases, cell property prediction can not only generate biological insight but also lead to actionable plans to benefit health and prevent diseases. The proposed single-cell property prediction methodology complements genetic screening^11–13^. While genetic screening works at the tissue and organ levels, cell property prediction works at the single-cell level. When coupled with single-cell RNA sequencing^14,15^ and single-cell proteomic^6^ analyses, our understanding towards cell biology can be greatly advanced.

Establishing the cell property predication capabilities at the single-cell level needs a unique set of technologies. Unlike most of the cell analysis methods that produce information at the endpoint, technologies enabling cell property prediction must keep the cells intact post-analysis so that these predictions can be verified and assessed^1,16^. To minimally affect cell properties, marker-free single-cell imaging appears to be a suitable technique^17–22^. It is also important to isolate each imaged cell and place it in an environment suitable for the cell to continue its development. The above requirements suggest that an imaging flow cytometer capable of producing marker-free 3D images of individual cells at high throughput is a desirable tool for this purpose^23^. Unlabeled or minimally labeled cells experience minimum unintended cell perturbations. The flow cytometer platform supports single-cell imaging, and 3D images provide rich information for the basis of cell property prediction. Artificial intelligence (AI) will be the cornerstone for cell property prediction. In our study, we chose the 3D imaging flow cytometer together with a position-tracked cell placement platform (CPP) for the hardware^24^ and a fused convolutional autoencoder-based classifier (CAE) architecture to make the cell property prediction^25,26^.

In our lab, we have developed a flow cytometer system capable of generating 3D single-cell images based on the design of a scanning light sheet and an array of pinholes in a configuration of a confocal microscope^20,23,25–27^. The system combines the high-throughput capabilities of flow cytometry and the imaging capabilities of microscopy. The setup supports 3D side scattering and fluorescent images, as well as 2D transmission images. As a marker-free method, the 3D side scattering image is formed by sectioning a cell into 10 slices and imaging the scattering centers on each of the slices, thus creating more information contents than 2D images. The data is then processed and interpreted by a machine learning model that deciphers complex patterns within the data and makes predictions of the cell properties. Finally, a cell dispensing platform that can track the cell image and the cell location for each single cell allows us to observe the cell behaviors over time and evaluate the accuracy of prediction^24^. To prove the concept, in this paper we perform two experiments, predictions of protein expression level^28–30^ and cell property changes after stresses. The latter also includes the ability to “detect” cell history (i.e. whether a cell experienced stress in the past).

In the first experiment, we try to predict protein expression levels of individual Chinese Hamster Ovary (CHO) cells. From the 3D scattering and 2D transmission images of GFP transfected CHO cells just harvested from the culture medium (i.e. day zero), without measuring the GFP expression level at the point of harvest, we can correctly predict, in most cases, those CHO cells that will later produce more GFPs than the rest of the population. We can predict the high GFP expression level group (i.e. top 10%) from the low and ordinary GFP expression level group at a balanced prediction accuracy of 88.2%.

In the second set of experiments, we divided the cells into two groups, cells that went through stresses and cells that did not. The experiment tried to determine whether a cell went through stresses (i.e. tell the cell history) as well as predict how the stressed cells would behave differently in the future. We performed a blind test in which the testers did not know whether the samples were stressed or not and hence the tester needed to not only predict whether the cell properties will change but also determine whether the cells imaged at “day zero” had the history of being stressed or not. We have applied two stressors to CHO cells: elevated temperature to 40 C and glucose deprivation. For the thermal-stressed group, a prediction balanced accuracy of 81.8% was achieved. For the glucose deprivation group, a prediction balanced accuracy of 99.4% was achieved. To evaluate the prediction accuracy, we harvested each group of cells 72 h after the prediction and ran these cells through a traditional flow cytometer.

## Results

### Prediction of cell protein expression level

The experiment contains three steps: sample preparation and data acquisition, training of the AI model, and prediction of cell protein expression level.

**Fig. 1:**
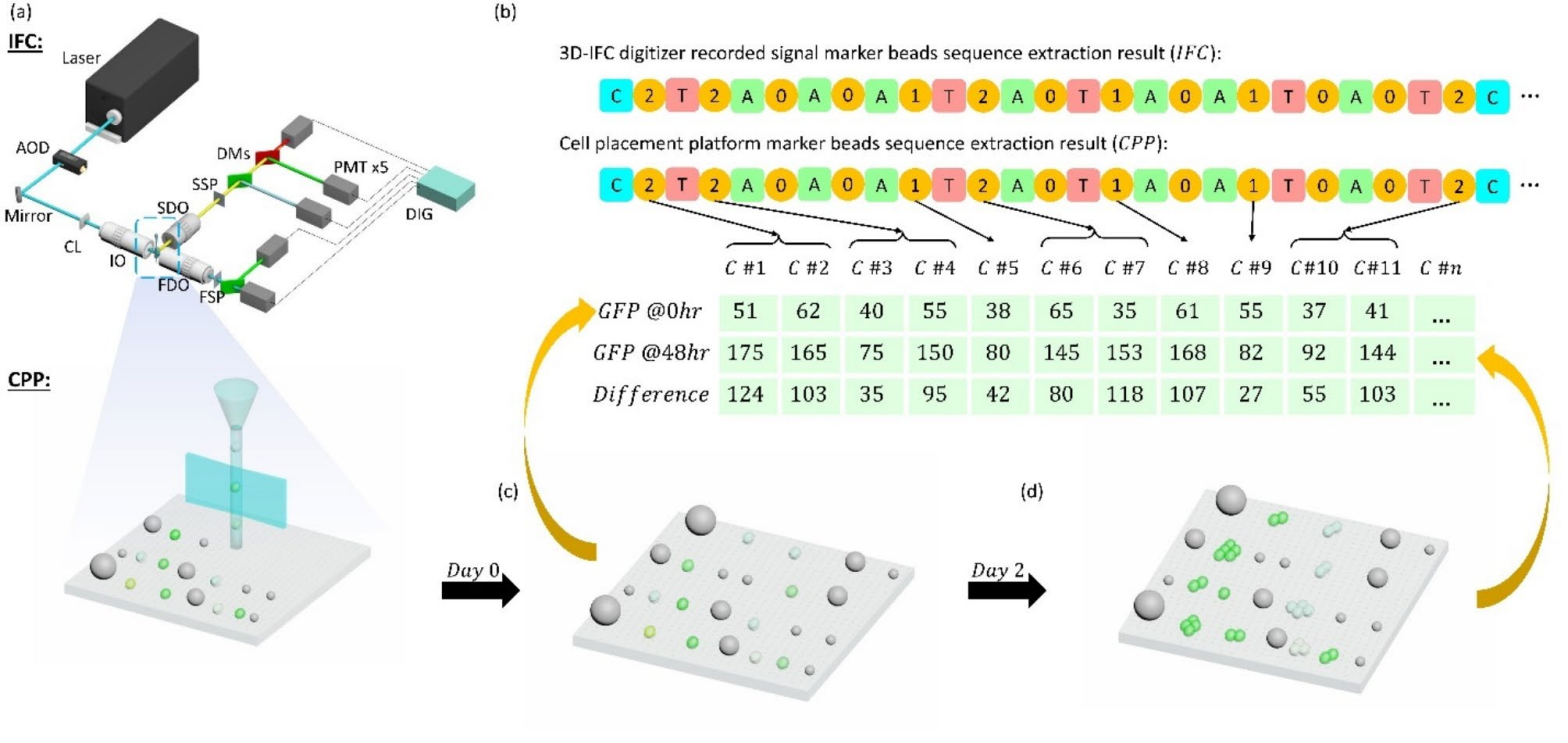
Methodology and workflow for prediction of CHO cell protein expression. (**a**) Cells and marker beads mixtures are examined by the 3D imaging flow cytometer (3D-IFC). Samples are then dispensed on the cell placement platform (CPP) in a first-in-first-out manner after exiting the 3D-IFC. (**b**) The order of cells and marker beads of different sizes or colors (represented here by A, T, C) from the 3D-IFC signals and CPP are compared for the purpose of matching the cell images to the cell positions and eliminating any sections showing deletion or displacement errors. An example of matched sequences is shown. Here we use a numerical number to represent the number of cells between two marker beads. For example, a sequence C/2/T represents that 2 cells exist between a marker bead C and a marker bead T. The sequence from the IFC should be identical to the sequence from the CPP if there are no deletion (i.e. missing cells) or displacement (e.g. cell position scrambling) errors. Any error sections will not be included in the analysis. The table below the IFC and CPP sequences shows the protein expression level of each single cell at 0 h and 48 h, and the difference in the value shows the protein production rate. (**c**, **d**) Schematic illustrations of what occur to each single cell on the CPP over time. Each cell is developed into a single-cell derived microcolony and produces more GFPs over time. Cells on the CPP are supplied with full growth media in an incubator at 37 °C with 5% $\:{CO}_{2}$. Legends in (**a**): AOD, acousto-optic deflector; CL, cylindrical lens; IO, 20×/0.42 illumination objective; SDO, 10×/0.28 side detection objective; SSP, side spatial filter; DMs, dichroic mirrors; FDO, forward detection objective; FSP, forward spatial filter; DIG,125 MSs1 digitizer; PMT, photomultiplier tube.

Our system comprises two hardware components that are interconnected: a 3D imaging flow cytometer and a robotic cell dispenser. The details of the hardware can be found in our previous publications^24^. The 3D-IFC system can produce 3D side scattering and fluorescent images as well as 2D transmission images of moving cells at a rate of 1000 cells/s. When the cells exit the system, they are dispensed in a first-in-first-out manner on a template together with the marker beads added to the sample to help register the cell location^24^. Figure 1a shows the overall schematic of the system. The maker beads sequences are used as references to relate the cell images to the cell locations on the cell placement membrane^24^.

CHO cells (GenTarget Inc., San Diego) that stably express humanized eGFP fluorescent protein was used^31,32^. After imaging and dispensing them on the CPP, the GFP protein expression level was measured by a fluorescence microscope at day zero (or 0 h). The cells were cultured on the membrane filter with a provision of full media in the incubator (with 37 °C, 5% $\:{CO}_{2}$) for 48 h. Figure 1b shows the sequence extraction results from both the IFC and CPP and the GFP FL protein expression level at 0 h and 48 h. An example of cells and beads on the CPP at 0 h and 48 h are shown in Fig. 1c,d.

**Fig. 2:**
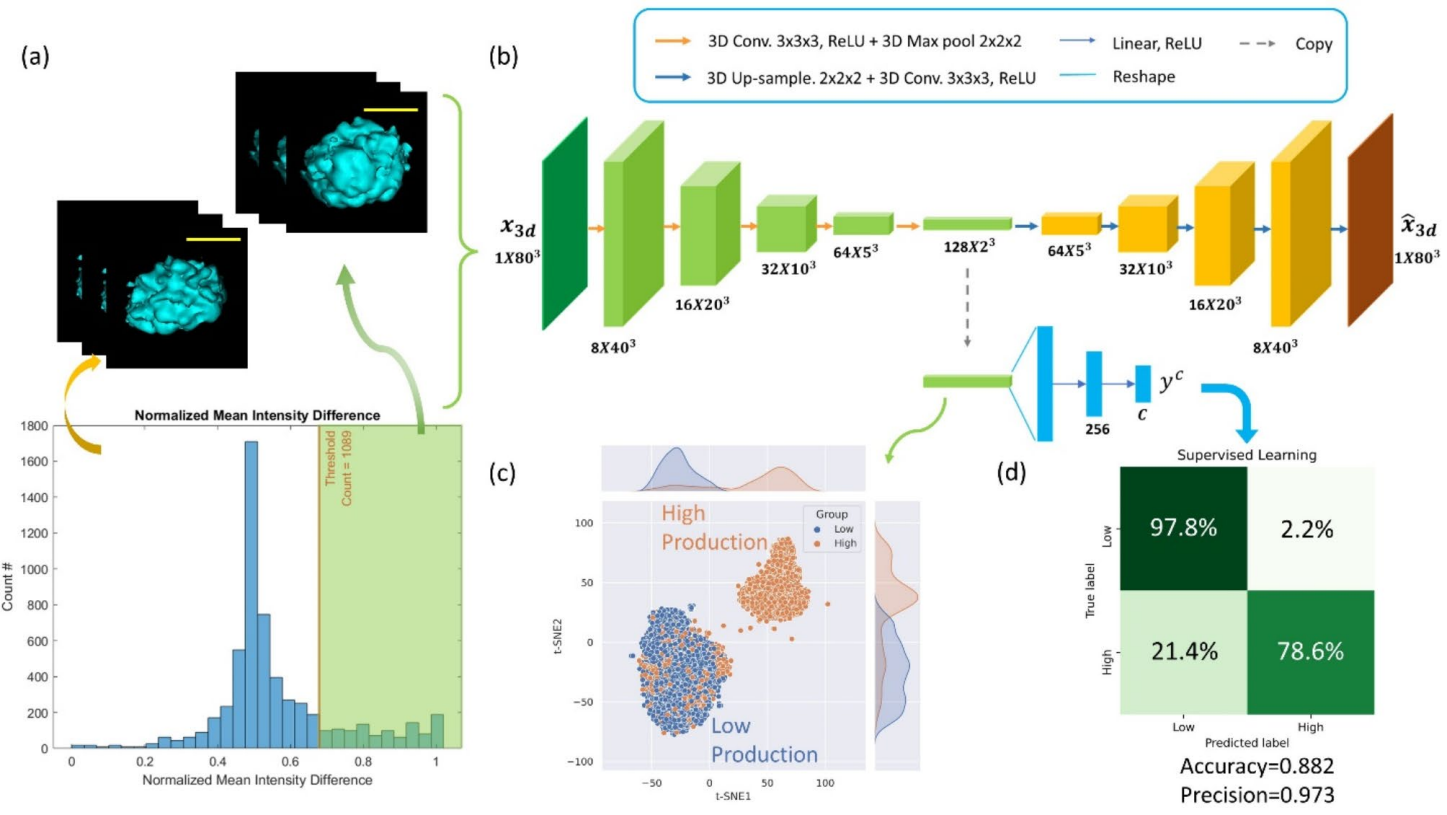
CHO cell protein expression level prediction from day zero cell images and machine learning. (**a**) From the histogram of GFP protein expression rate, the high production group and low production group are gated out. Representative 3D SSC images from each group are shown. (**b**) The machine learning model used to analyze and classify the 3D SSC images. Through the training process, the model learns to classify high GFP production cells and low/ordinary GFP production cells using the 3D SSC images captured at 0-hour. (**c**) Visualization of the latent encoded features from the trained convolutional autoencoder. (**d**) Confusion matrix of the machine learning model for performance evaluation. A balanced accuracy of 88.2% was achieved in prediction of single cell protein production capability.

From these data, the relative GFP protein production rate of each single cell over the past 48 h was calculated. Based on the histogram of protein production rate, a simple gating was applied to separate the high (top 10%) expression level group from the rest of the population. Figure 2a shows the histogram of the protein expression from single cells and some representative 3D SSC images in each group. We use 3D SSC images as inputs for a machine learning model, which is a convolutional autoencoder-based classifier (CAE), to train the neural network. The results provide the “ground truth” of the GFP generation rate of each CHO cell. Figure 2b shows the network architecture.

We use 1000 cell images to train the ML model to classify the CHO cells into the high GFP production group and the low/ordinary GFP production group in a manner of supervised learning. The images used for training were unlabeled 3D SSC and 2D transmission images taken from the 3D-IFC at 0 h (i.e. at day zero) before the 48-hour culture.

Through the training process, the network encodes the input images to the latent convolutional layer. During inferencing, the network uses the flattened latent channels to make the prediction. Figure 2c,d show the t-SNE visualization result and the corresponding confusion matrix of the inferencing results that predict which group each GFP-transfected CHO belongs to. The classification report shows a prediction accuracy rate of 88.2%. To our best knowledge, this is the first demonstration of predicting the protein production rate of individual cells without labeling, enabled by the unique combination of hardware and machine learning.

## Prediction of cell property variations after stress

Predicting cell property changes caused by the cell history can provide new insight for disease development, valuable for preventive medicine^2,33,34^. In the realm of drug response and cell therapy, predicting cellular properties days or weeks before the responses actually happen can inform early selections of target cells and possibly lead to the design of more effective treatments^35^.

In our experiment to investigate this concept, we had CHO cells undergo two stresses, thermal stress and nutrient deprivation, for a relatively short time period not to obviously affect the cell’s health or viability. We took marker-free images of the stressed and unstressed cells before any apparent phenotypical changes occurred and used the images taken at day zero to predict whether the cells being analyzed would eventually show the effect of stress.

**Fig. 3:**
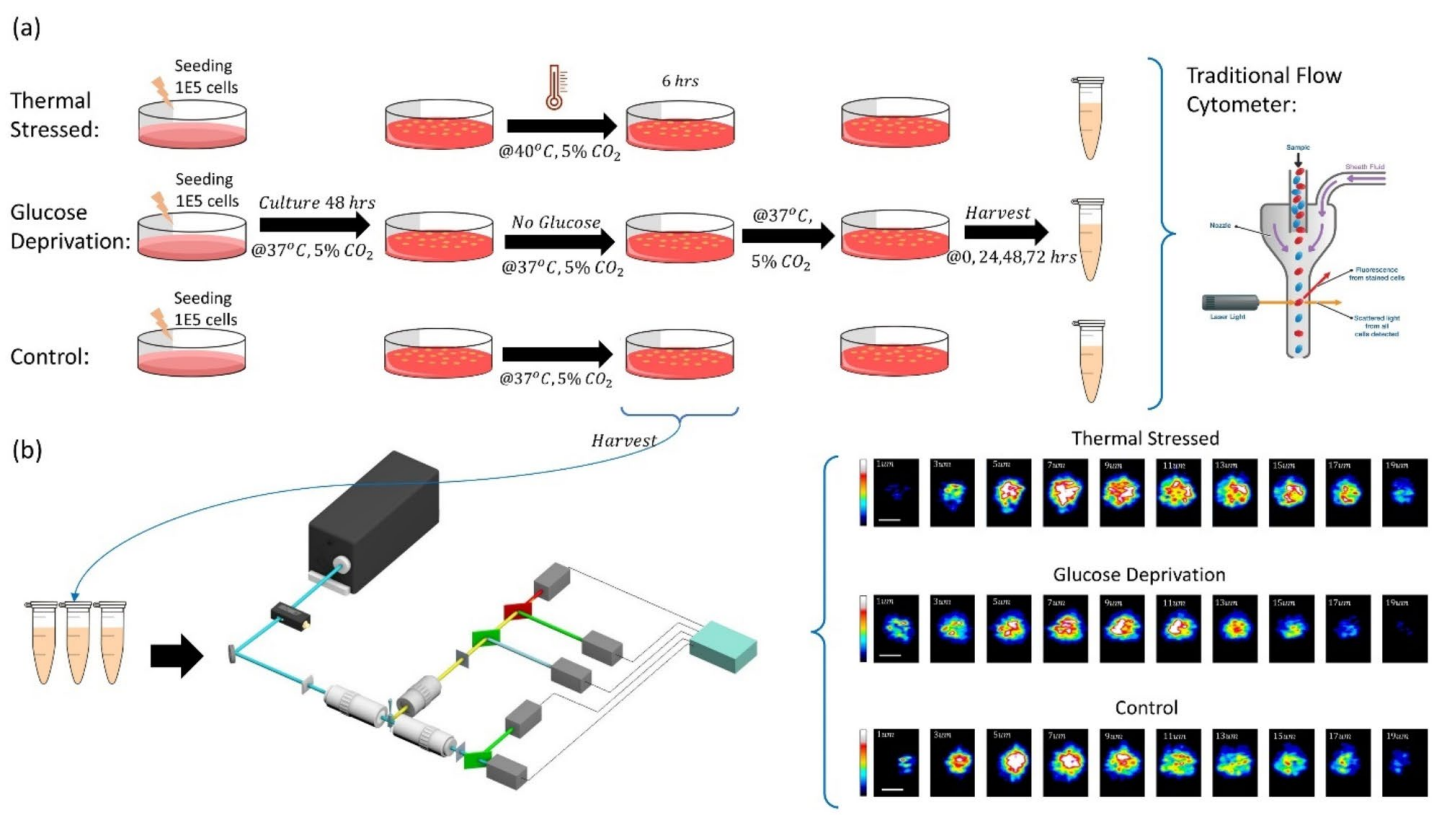
Overall workflow for prediction of cell property changes after experiencing stresses. (**a**) Flow chart for cell culturing and stress introduction. The stressed and control samples are examined by a commercial flow cytometer. (**b**) After 6 h of stress treatments, cells are harvested and examined using the 3D-IFC (i.e. day zero) and the 3D SSC images were used to predict the cell behaviors using an ML model. Also shown are representative single cell images from stressed and control groups. Each 3D cell image is sectioned into 10 cross sections showing the scattering patterns of each cross-section.

Figure 3 shows the overall workflow of the experiment. As shown in Fig. 3a, forty-eight hours prior to the stress treatment, 3 dishes of CHO cells were seeded with an initial concentration of $\:{10}^{5}/mL$ cells. After 48 h of culturing in a 37 °C, $\:\:5\%\:{CO}_{2}\:$incubator supplemented with full culture media, cells were in the logarithmic growth phase. One dish of cells was transferred to an incubator in 40 °C, $\:\:5\%\:{CO}_{2}$ to thermally stress the cells. The other dish of cells was supplied with the culture media without glucose. Another dish was used as a control. Both the control dish and the glucose-deprived dish were placed in a 37 °C, $\:\:5\%\:{CO}_{2}\:$incubator. After 6 h, cells from all three dishes were harvested and examined by the 3D IFC to generate “day zero” images. Figure 3b shows examples of 3D SSC single cell images having each cell image sectioned into 10 cross sections. Then all cells were cultured in a 37 °C, $\:\:5\%\:{CO}_{2}$ incubator in full culture media. Each group of cells was then examined using forward scattering (FSC) and back scattering (BSC) signals of a commercial flow cytometer (WOLF system, NanoCellect Biomedical Inc.) at a 24-hour interval and up to 72 h to monitor any morphological changes for stressed cells.

Supplementary Figure S1 and Figure S2 show the comparison of the FSC and BSC signals from a commercial flow cytometer between the control group and the glucose-deprived and thermal-stressed groups at 0 h and 72 h. At 0 h when the cells just went through the stresses, hardly any difference among different cell groups could be detected by a commercial flow cytometer. After 72 h, a modest shift in the histograms of FSC and BSC signals developed for the glucose-deprived cells, and the changes in signal distribution were even smaller for thermal-stressed cells relative to the control group. The results indicate that even with continuous monitoring of the cell properties for 72 h, it is very hard to tell whether an individual cell has gone through stresses or produced stress-induced morphological changes using a commercial flow cytometer without labelling of specific biomarkers. What we want to investigate is whether it is possible to detect a cell’s history (i.e. stressed or not) and predict its future morphological changes using 3D marker-free cell images and machine learning.

**Fig. 4:**
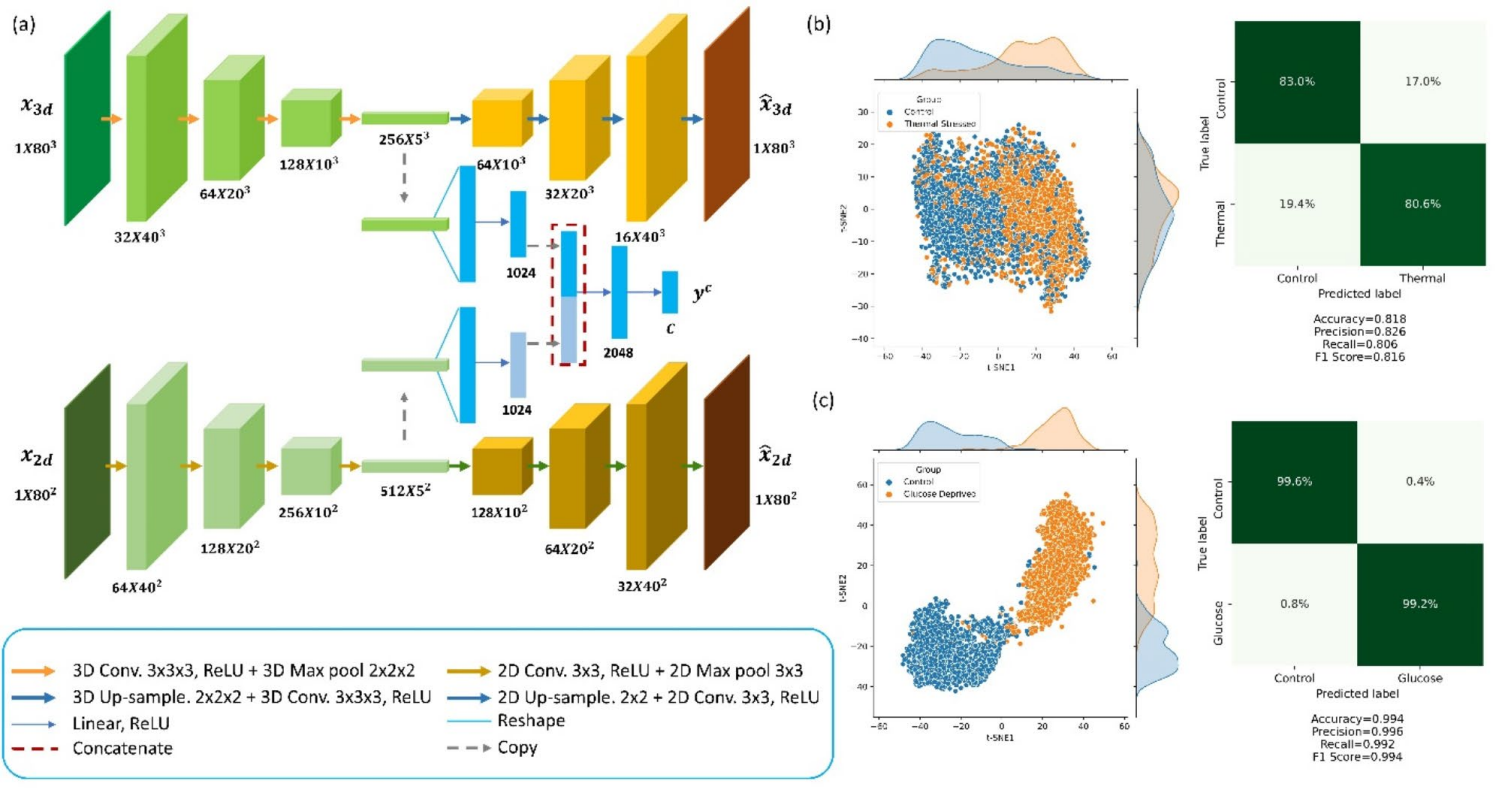
Detection of cell history and prediction of post-stress cell property variations. (**a**) The fused convolutional autoencoder (CAE) based classifier combines two CAE structures, each taking a single modality image as input. In the latent space, the latent features are concatenated and used to make the prediction. (**b**) Visualization of the latent encoded features from the trained fused CAE-based classifier for the thermal-stressed group. The ability of detection of cell history (experienced thermal stress or not) is summarized in the Confusion Matrix, showing a balanced accuracy of 81.6%. (**c**) Visualization of the latent encoded features from the trained fused CAE-based classifier for the glucose-deprived group. The ability of detection of cell history (experienced glucose deprivation stress or not) is summarized in the Confusion Matrix, showing a balanced accuracy of 99.4%.

We used a fused convolutional autoencoder that combines 2D CAE and 3D CAE, each taking a single image modality as the input, to detect cell (stress) history and predict cell properties post stress. The convolutional layers extract image features, while the max-pooling kernels encode them to the subsequent layers during the contracting paths. The latent spaces from the two CAEs are combined by concatenation, followed by a fully connected layer and a Softmax layer to make a classification decision. Figure 4a shows the network structure.

We conducted experiments to detect whether a cell was stressed and predict its future morphological changes for both glucose deprived and thermally stressed groups using the cell images taken at “day zero” and the fused CAE model. The results of the thermally stressed and glucose-deprived groups are shown in Fig. 4b,c, respectively. The confusion matrices show that the fused CAE model was able to accurately detect the history of the cells and predict cell property changes for the stressed cells at day zero. The fused CAE demonstrates an averaged balanced prediction $\:{F}_{1}$ score of 0.994 for the glucose-deprived cells across all the Cross Validation (CV) folds. The prediction of cell property changes for thermally stressed cells gives rise to an averaged balanced prediction $\:{F}_{1}$ score of 0.814 for all CV folds.

## Methods and materials

### Preparation of CHO cells for protein expression prediction

CHO cell line (GenTarget Inc., San Diego) that stably expresses the humanized eGFP fluorescent protein was used for the protein expression level prediction experiment. Cell lines were cultured with growth media (Ham’s F12K (Kaighn’s) Medium, 10% Fetal Bovine Serum, 1% Antibiotic-antimycotic) in a 10 cm petri dish to 90% confluency before harvesting. After culturing, cells were harvested and resuspended to a concentration of ~ 5 × 10^5^ cells/mL in 1X PBS. Prior to running through the 3D-IFC, cells were mixed with marker beads (10 μm, 20 μm, 30 μm) at a cell-to-bead ratio of around 2:1. After dispensing the cells and the marker beads on the cell placement platform (CPP), cells were cultured on the membrane filter in growth media. GFP protein expression level of each cell was registered at 0 h and 48 h using a fluorescence microscope.

### Preparation of CHO cells for detection of cell stress and prediction of post-stress cell property changes

Depending on cell concentration, the growth curve of CHO cells normally consists of a logarithmic cell proliferation phase, a stationary phase, and a death phase. Cells normally enter the exponential cell proliferation phase 24 to 72 h after seeding. In this experiment, 48 h prior to the stress treatments, 3 dishes of CHO cells were seeded with an initial concentration of $\:{10}^{5}/mL$ cells. After 48 h of culturing in a 37 °C, $\:\:5\%\:{CO}_{2}\:$incubator supplemented with full growth media (DMEM with 25mM D-Glucose, 10% Fetal Bovine Serum, 1% Antibiotic-antimycotic), cells entered the logarithmic growth phase. One dish of cells was transferred to an incubator in 40 °C, $\:\:5\%\:{CO}_{2}$ for thermal stress. Another dish of cells was supplemented with the culture media without glucose (DMEM no Glucose, 10% Fetal Bovine Serum, 1% Antibiotic-antimycotic). The other dish was used for the control sample. The glucose deprived dish and the control dish were cultured in a 37 °C, $\:\:5\%\:{CO}_{2}\:$incubator. After 6 h of incubation for thermal stress and glucose deprivation for two of the three dishes, cells were harvested and resuspended to a concentration of ~ 5 × 10^5^ cells/mL in 1X PBS.

### Customized CAE-based classifier for image classifications

A deep autoencoder (DAE) typically includes an encoder and a decoder, each comprising two fully connected layers stacked together^36^. In contrast to a DAE, the deep convolutional autoencoder (DCAE) integrates convolutional and deconvolutional layers as part of both the encoder and decoder^37^, instead of solely relying on fully connected layers. This approach is aimed at leveraging the benefits of a convolutional neural network for image processing, particularly for 3D image processing^38,39^. Convolutional Neural Networks exhibit distinguished properties such as sparse interactions, parameter sharing, and equivariant representations, which are crucial for effectively translating latent features.

In this paper, two CAE-based classifiers are developed, the 3D CAE-based classifier and the fused CAE-based classifier. The fused CAE-based classifier combines two CAE structures (2D CAE and 3D CAE), each taking a single image modality as the input. The convolutional layers extract image features, while the max-pooling kernels encode them to the subsequent layers during the contracting paths. The latent space from the two CAEs is combined by concatenation and followed by a fully connected layer and a Softmax layer to enable a classification decision. The reconstructed images output from the decoders are leveraged to optimize the loss function during the model training process. The architecture of the fused CAE-based classifier is illustrated in Fig. 4a. The 3D CAE-based classifier can be considered as part of the fused network, while the concatenation in the latent space is eliminated. The output of the Softmax layer can be written as:

1 $$\:{\widehat{\varvec{y}}}_{\varvec{i}}=\frac{{e}^{{\varvec{x}}_{\varvec{i}}}}{{\sum\:}_{j=1}^{C}{e}^{{\varvec{x}}_{j}}},i=\text{1,2},\dots\:,C$$

where $\:\varvec{x}$ is the input vectors, C is the number of classes.

For the first 20 epochs of training, the CAE-based classifier was trained to minimize the mini-batch averaged mean-square error (MSE) loss between the input images and generated output images. The fused CAE-based classifier was optimized using a weighted MSE loss including the contributions of 2D and 3D CAE. The mini-batch averaged mean-square error loss can be expressed as:

2 $$\:{L}_{MSE}={w}_{1}{L}_{MSE,2d}+\left(1-{w}_{1}\right){L}_{MSE,3d}=\frac{1}{N}\sum\:_{j=1}^{N}\left(\frac{{w}_{1}}{{M}_{2d}}{\sum\:}_{i=1}^{{M}_{2d}}{\left({x}_{2d,i,j}-{\widehat{x}}_{2d,i,j}\right)}^{2}+\frac{1-{w}_{1}}{{M}_{3d}}{\sum\:}_{i=1}^{{M}_{3d}}{\left({x}_{3d,i,j}-{\widehat{x}}_{3d,i,j}\right)}^{2}\right)$$

where $\:{x}_{2d}$ and $\:{\widehat{x}}_{2d}$ are the input 2D transmission image and model-generated image, respectively. $\:{M}_{2d}$ is the flattened transmission image vector dimension, $\:{x}_{3d}$ and $\:{\widehat{x}}_{3d}$ are the input 3D SSC image and model-generated image, respectively. $\:{M}_{3d}$ is the flattened SSC image vector dimension. $\:N$ is the batch size in the mini-batch. $\:{w}_{1}$ is the mean-square-error loss weight assigned to the two image modalities. In the 3D-CAE based classifier, $\:{w}_{1}=0$ since there is no 2D image input.

As another component in the overall loss function, the averaged cross-entropy loss can be written as

3 $$\:{L}_{CE}=-\frac{1}{N}\sum\:_{i=1}^{N}{\varvec{y}}_{\varvec{i}}\left(\varvec{x}\right)\cdot\:\text{log}\left({\widehat{\varvec{y}}}_{\varvec{i}}\left(\varvec{x}\right)\right)$$

where $\:{\varvec{y}}_{\varvec{i}}$ is the ground truth class vector, $\:{\widehat{\varvec{y}}}_{\varvec{i}}$ is the predicted class vector, and $\:N$ is the data size in the mini-batch.

The final weighted loss function, defined in (4), was adopted to train the network.

4 $$\:L={{w}_{2}\cdot\:L}_{CE}+\left(1-{w}_{2}\right)\cdot\:{L}_{MSE}$$

where $\:{w}_{2}$ is the weight coefficient for the cross-entropy term relative to the MSE loss.

All deep learning models are implemented in the PyTorch framework and trained on an 8-core machine with Intel^®^ core™ i7-11700 K processor and NVIDIA GeForce RTX 3080 Ti with 12 GB of VRAM. Both models were trained for 150 epochs using the Adam optimization algorithm with a batch size of 4. To ensure robust performance evaluation, a stratified 5-fold cross-validation was employed.

## Discussion

In this study, we have investigated the novel idea of predicting cell properties for single cells. This is, by far, the first experiment of this kind and the results, although preliminary, have been promising. To pursue this path, the technologies need to satisfy the following requirements: analysis with minimum disturbances to the cells (i.e. favoring label-free detection), high throughput, high information content, and an ability to match the cell analysis data to the cell location for assessment of the prediction results. Fortunately, the development of 3D imaging flow cytometer, the robotically controlled cell placement platform, and a fused CAE neural network meet the above requirements, thus enabling the experimental study of cell fate prediction. As the technology advances and more studies in this area become available, the concept and method will lead to a deeper understanding of cell biology and find applications in drug production and preventive medicine. In the longer future, the ability to predict cell fate might also contribute to the development of cell therapy and regenerative medicine.

The overall workflow of cell property prediction can capture and integrate information from multiple cellular features, such as morphology, signaling pathways, and gene expression, at different time points during cellular development. This integration provides a comprehensive understanding of the regulatory mechanisms and molecular events that govern cell properties and behaviors. The current approach uses supervised ML that relies on the ground truth labels. A natural next step is to use unsupervised learning or self-supervised learning, which simplifies the network training and offers higher probabilities of discovering novel cellular states.

Despite the promising results demonstrated in this study, several challenges and limitations remain to be addressed. The inability of the current system to obtain 3D small angle scattering images limits the amount of useful information one can use to predict cell properties. Moreover, the interpretability of the AI network in a biological context requires further research.

In conclusion, our study demonstrates the feasibility of cell property prediction. The encouraging initial results such as 88% accuracy for the prediction of high protein production cells, 82% and 99% accuracy for detection of cell stress history and prediction of post-stress cell properties pave the way for future explorations in this new area of research.

## Electronic supplementary material

Below is the link to the electronic supplementary material.

Supplementary Material 1

## Data Availability

The PyTorch codes that support this paper have been uploaded to a public GitHub repository: https://github.com/ZunmingZhang/Cell-Properties-Prediction-using-3D-IFC.git.
