# Supplementary material for: Predicting cell properties with AI from 3D imaging flow cytometer data
Source: Sci Rep. 2025 Feb 17;15:5715. doi: 10.1038/s41598-024-80722-6 (PMC11833109; doi:10.1038/s41598-024-80722-6)
Supplement: Supplementary file 1 — Supplementary Material 1 [file 41598_2024_80722_MOESM1_ESM.docx]

**Predicting Cell Properties with AI from 3D Imaging Flow Cytometer Data**

Zunming Zhang^1^, Yuxuan Zhu^1^, Zhaoyu Lai^1^, Minhong Zhou^1^, Xinyu Chen^1^, Rui Tang^2^, William Alaynick^2^, Sung Hwan Cho^2^, Yu-Hwa Lo^1, *^

^1^ Department of Electrical and Computer Engineering, University of California, San Diego, La Jolla, California, 92093, USA;

^2^NanoCellect Biomedical Inc., San Diego, California, 92121, USA;

* Author to whom correspondence should be addressed.

**Supplementary Figures**


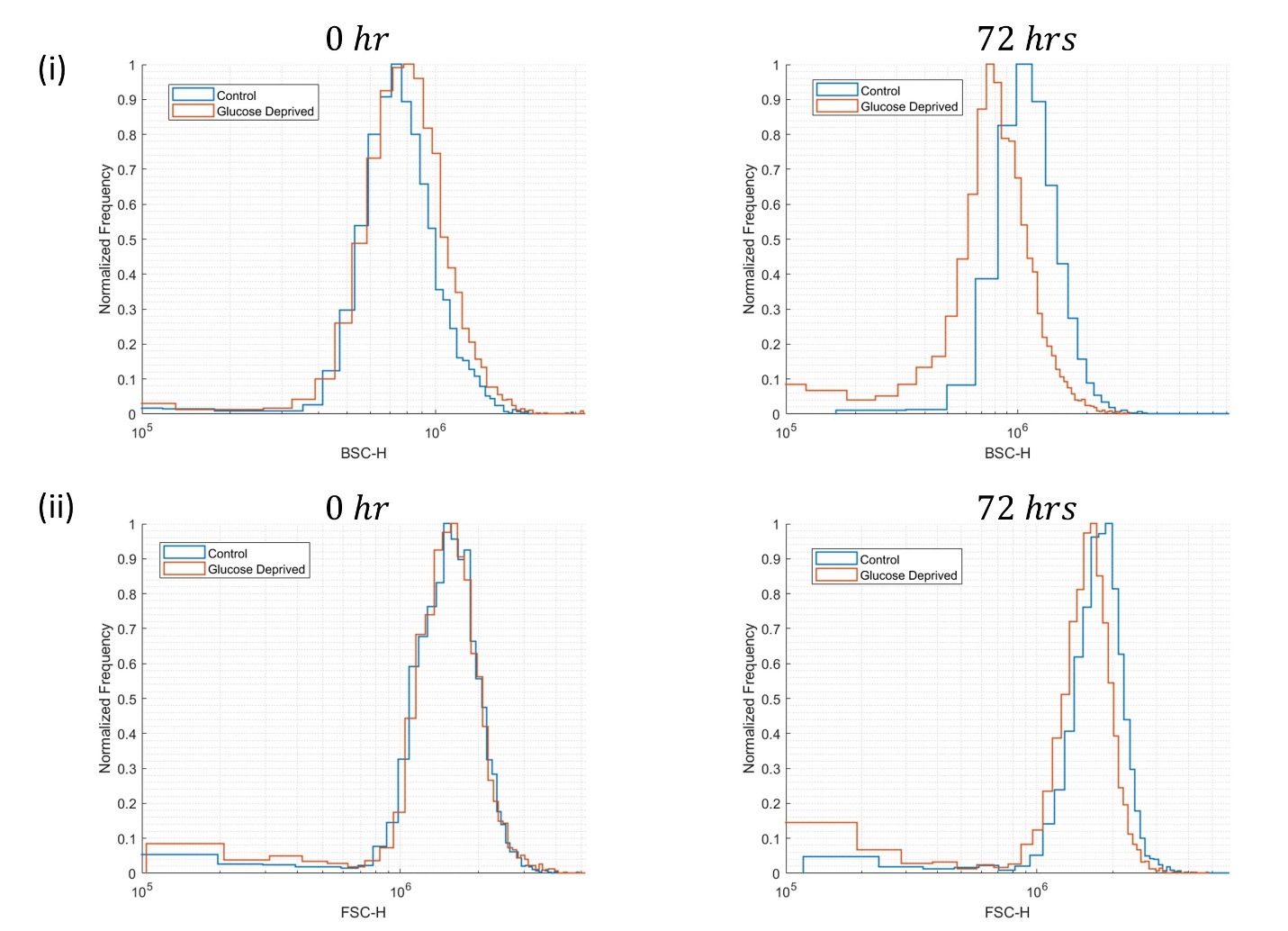


**Figure S1.** Results of a commercial flow cytometer for glucose deprived group in comparison with the control group at 0-hour and 72-hour after stress. Back scattering (i) and forward scattering (ii) signals from glucose deprived cells at 0hr and 72 hours. A modest shift of signal distribution for glucose deprived cells appears 72 hours after stress.


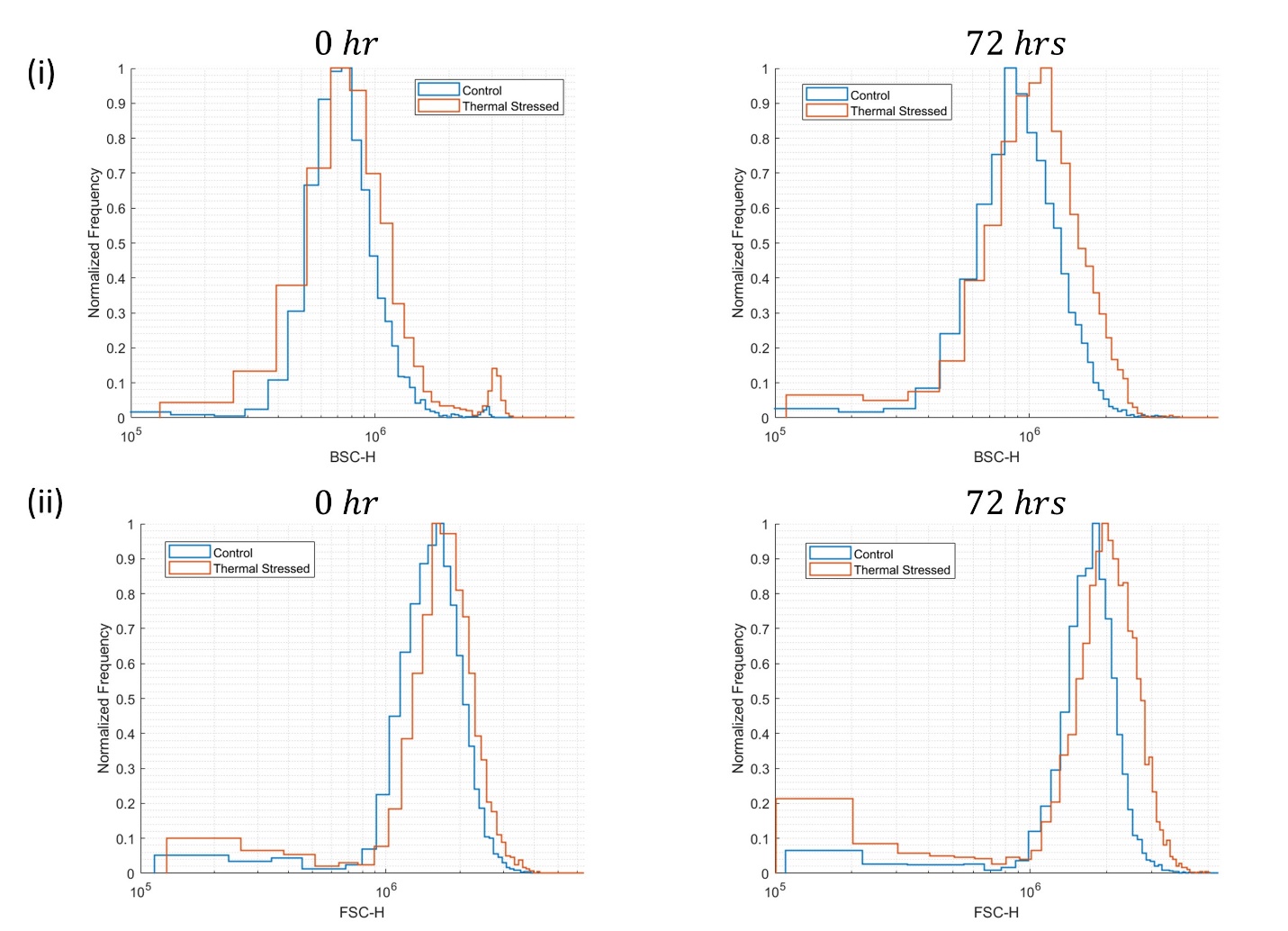


**Figure S2.** Results of a commercial flow cytometer for thermal stressed group in comparison with the control group at 0-hour and 72-hour after stress. Back scattering (i) and forward scattering (ii) signals from thermal-stressed cells at 0hr and 72 hours. 72-hour. The signal shift for thermal-stressed cells is not obvious.

The results in Figure S1 and Figure S2 indicate that commercial flow cytometers are unable to detect cell morphological changes right after stress without biomarker labelling. At 72 hours, the results confirm distinct cell fates across the different treatment groups.
